# Supplementary figures and images for: Germline cis variant determines epigenetic regulation of the anti-cancer drug metabolism gene dihydropyrimidine dehydrogenase (DPYD)
Source: eLife. 2024 Apr 30;13:RP94075. doi: 10.7554/eLife.94075 (PMC11060711; doi:10.7554/eLife.94075)

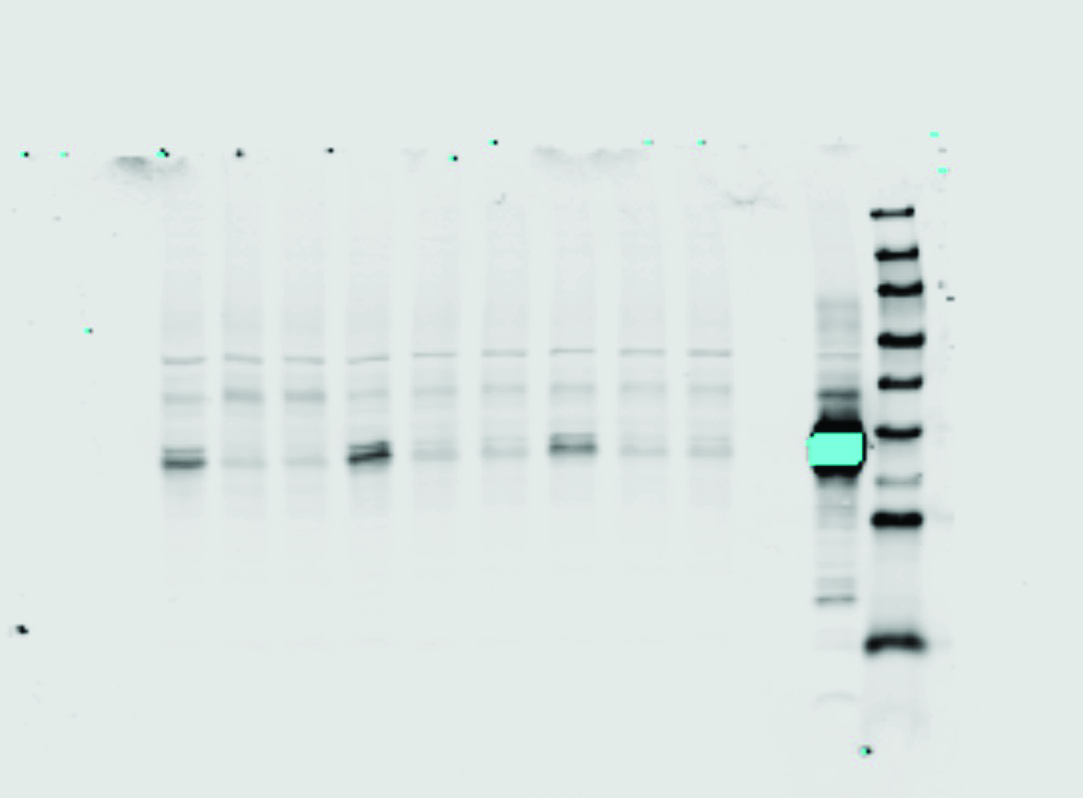

Supplement: Figure 6—source data 1. [file elife-94075-fig6-data1.tif]

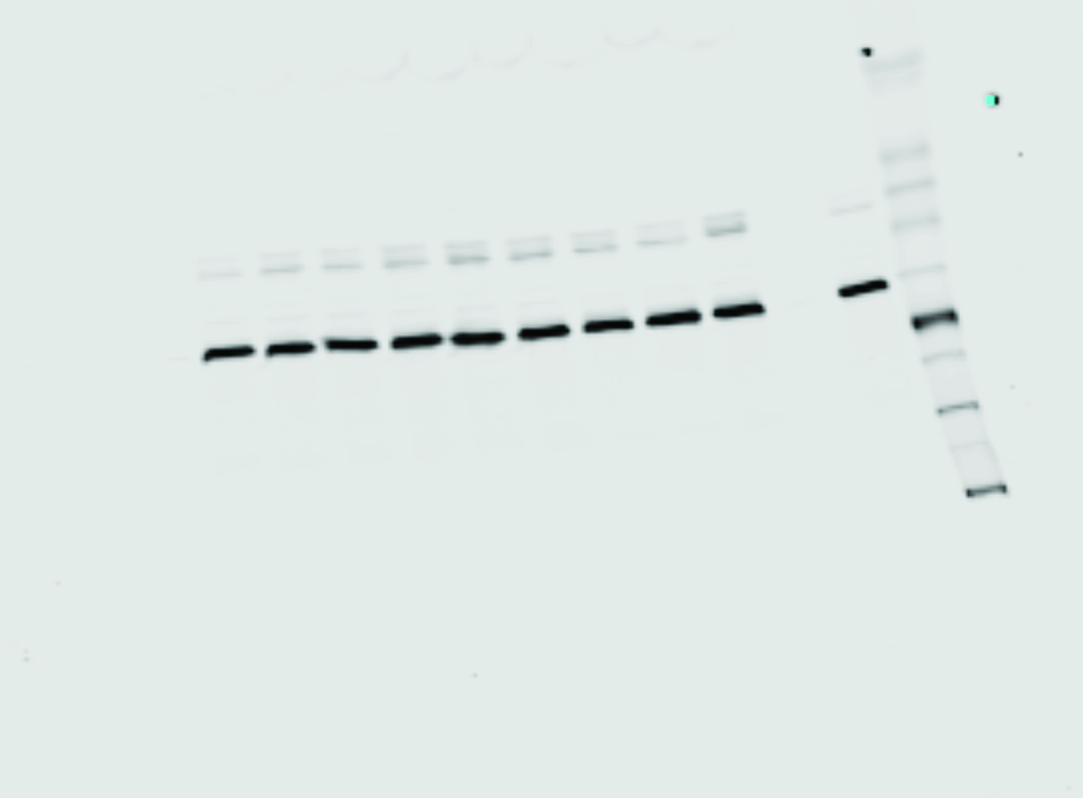

Supplement: Figure 6—source data 2. [file elife-94075-fig6-data2.tif]

**A**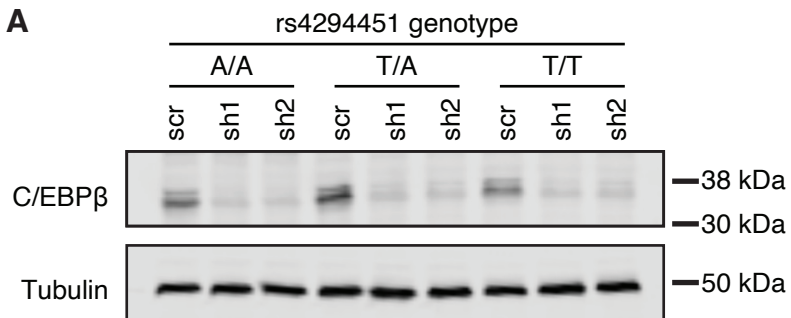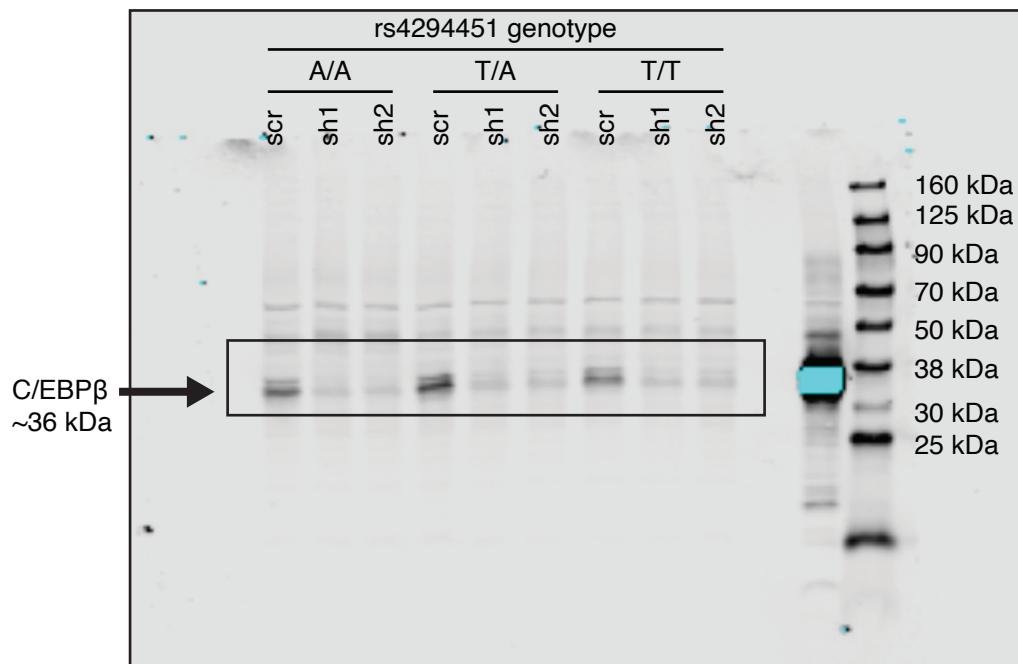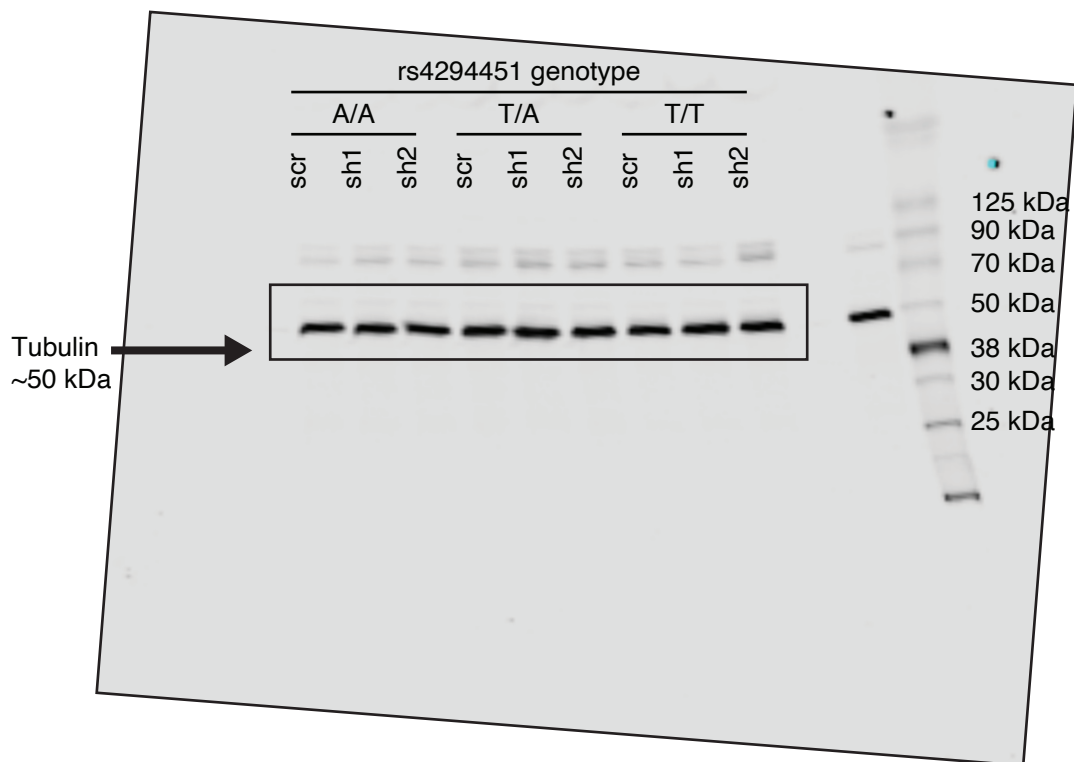

Image rotated -4.5° from scanned original for use in Figure 6A.

Supplement: Figure 6—source data 3. [file elife-94075-fig6-data3.pdf]
