# Supplementary material for: Germline cis variant determines epigenetic regulation of the anti-cancer drug metabolism gene dihydropyrimidine dehydrogenase (DPYD)
Source: eLife. 2024 Apr 30;13:RP94075. doi: 10.7554/eLife.94075 (PMC11060711; doi:10.7554/eLife.94075)
Supplement: Supplementary file 1. [file elife-94075-supp1.docx]

| **Supplementary File 1: Primers used for ChIP, cloning, qPCR, and site-directed mutagenesis.** | | |
| --- | --- | --- |
| **Target** | **Forward Primer (5’-3’)** | **Reverse Primer (5’-3’)** |
| E9 ChIP-1 | CCTACCTACCACCCCCAAGA | TGCCCAGGACATTACACATGA |
| E9 ChIP-2 | CATCAGGTTGCTTTTTGCAGC | TTAGCGTGGACTACCAGGGA |
| E9 ChIP-3 | TGGGACTCAAAAAGCGGTTCA | GGTGTTGCGGGTGCTGATTA |
| E9 ChIP-4 | GGTTCAACAGAGGATGCAACAC | AGGGAAAGATTTTCCTGGCCC |
| E9 ChIP-5 | TAAAGATGCACCGAGGTGGG | AGTGTCTGGATTTAAGTAGATGTGC |
| E9 ChIP-6 | AGCAGGAATGAGAAGGAGAGAAG | TCAGTCTCTCACTCCAAACCC |
| E9 ChIP-7 | GCTCTTTTCATTGAAGCCTAAAACA | CCATCATACTTTTTCCAATTGTTGC |
| E9 ChIP-8 | AATGATGAGAGGAAGATGACAAAGT | TGCCTACGCGATGAGTTGTA |
| DPYD promoter | AGTCGATATCCACAGTGTCTGTGTCTGGC | AGTCAAGCTTGCTCGATGTCCGCCGAG |
| E9 region | AGTCGGTACCGAATAAAACCAAAATAAAATCCATTTGGACGTTT | AGTCGAGCTCTTTGTGCAAAGGACCTTGGTATTTCC |
| Rs4294451 A>T | AAAGAAAAATAAATAAAAAAAGGAAAAATCTATAAGC | TTCTGGGGGTTGGTGTTG |
| Cas9 | AACAGCCGCGAGAGAATGAA | CACGGGGTGTTCTTTCAGGA |
| DPYD | GTAAGGACTCGGCGGACATC | GCCGAAGTGGAACACAGAGT |
| L32 | CCTTGTGAAGCCCAAGATCG | TGCCGGATGAACTTCTTGGT |
| CEBPB | CGCCGCCTGCCTTTAAATC | AAGCAGTCCGCCTCGTAGTA |
