# Supplementary material for: Germline cis variant determines epigenetic regulation of the anti-cancer drug metabolism gene dihydropyrimidine dehydrogenase (DPYD)
Source: eLife. 2024 Apr 30;13:RP94075. doi: 10.7554/eLife.94075 (PMC11060711; doi:10.7554/eLife.94075)
Supplement: Supplementary file 2. [file elife-94075-supp2.docx]

| **Supplementary File 2. Sequences and positions of the primers used for 3C analysis.** | |
| --- | --- |
| **Primer position (TSS: +1)** | **Sequences (5’-3’)** |
| -22822 | GGGAAGTTGAGAGAGCTAGGC |
| -19322 | TGCTCTGTCAGCTGAGAAGACCTAGA |
| -16420 | GTCACTACTGGGACTCTGAGAAA |
| -15616 | AAAAGAAATTGCAACCTCTGGCA |
| -11097 | GTTGCTTTTTGCAGCTGGGAT |
| -8087 (also used as anchor for E9 region) | AGTGCTTGAAGCTGATGAAGGG |
| -5884 | CTGCAGAACAAGAACAGCACAT |
| 1280 (also used as anchor for promoter) | TTAGGGTAGTCTATTCCTTTTTGGT |
| 2826 | TGCTTTGTGAGTGTACTGTTTGG |
| 3076 | CCTCCACCGGCAAGGATAAT |
